# The impact of COVID-19 pandemic restrictions on physical activity and mental health status of Iranian people

**DOI:** 10.1186/s13102-022-00584-1

**Published:** 2022-10-29

**Authors:** Esmaeel Saemi, Hadi Nobari, Georgian Badicu, Habibollah Ghazizadeh, Ali Pashabadi, Fatemeh Imani, Filipe Manuel Clemente, Ana Filipa Silva, Sana Afrash

**Affiliations:** 1 Department of Motor Behavior and Sport Psychology, Faculty of Sport Sciences, Shahid Chamran University of Ahvaz, Ahvaz, 6135783151 Iran; 2 Department of Exercise Physiology, Faculty of Educational Sciences and Psychology, University of Mohaghegh Ardabili, Ardabil, 56199-11367 Iran; 3 Faculty of Sport Sciences, University of Extremadura, 10003 Cáceres, Spain; 4 Department of Physical Education and Special Motricity, Faculty of Physical Education and Mountain Sports, Transilvania University of Braşov, 500068 Braşov, Romania; 5 Department of Motor Behavior, Faculty of Sport Sciences, Kharazmi University, Tehran, 1571914911 Iran; 6 Department of Sport Biological Sciences, Faculty of Sport Sciences and Health, Shahid Beheshti University, Tehran, 1983969411 Iran; 7 Escola Superior Desporto e Lazer, Instituto Politécnico de Viana do Castelo, Rua Escola Industrial e Comercial de Nun’Álvares, 4900-347 Viana do Castelo, Portugal

**Keywords:** COVID-19, Physical activity, Self-esteem, Social physical anxiety, Iran

## Abstract

**Background:**

Coronavirus-19 (COVID-19) is a highly contagious disease caused by acute respiratory syndrome that can negatively impact physical activity as well as mental health of people in the world. Since Iran is one of the countries deeply affected by the COVID-19 pandemic, therefore, the aim of this study was to investigate the impact of the COVID-19 pandemic restrictions on physical activity behaviours, as well as on mental health indicators among Iranian people.

**Methods:**

In this cross-sectional study, 335 participants were included (155 males, mean age = 30.06 ± 14.58 years). Participants were selected through the convenience sampling from different parts of Iran mainly through virtual social networks and filled out an electronic questionnaire in the form of Google Form online survey measuring physical activity behaviours (The International Physical Activity Questionnaire; IPAQ; Short Form) and two mental health indicators–self-esteem (Rosenberg self-esteem scale) and social physical anxiety (*7*-*items social* physique *anxiety scale*). The questionnaires were provided to the participants in the spring of 2021 for 15 days and they were asked to answer it in two periods before the outbreak of coronavirus (winter 2020) and the period during the outbreak of coronavirus (spring 2021).

**Results:**

The results showed that participants reported significant differences from before to during the pandemic in all three variables of physical activity (significant decreases), self-esteem (significant decreases), and social physical anxiety (significant increases) regardless of participants’ gender. The results of correlation test between changes in physical activity and changes in mental health indicators showed that COVID-19 pandemic negatively impacted self-esteem and social physical anxiety. However, we have not found any significant association of physical activity’s change with social physical anxiety or self-esteem’s changes before and during the pandemic.

**Conclusions:**

The findings of the present study indicate a detrimental effect of the COVID-19 pandemic on the physical activity and mental health among Iranian people. Public policies aiming to buffer the negative impact on COVID-19 Iranians’ health are urgently needed.

## Background

Coronavirus-19 (COVID-19) is a highly contagious disease caused by acute respiratory syndrome [1]. The disease was first observed in mid-December 2019 in Wuhan, China. Since the outbreak of the virus, the number of reported cases has increased rapidly and has affected the whole world. On January 30, 2020, the World Health Organization (WHO) affirmed the disease as “an international public health emergency” [2]. By the end of July 2021, more than 192 million people worldwide has been infected by the disease, of which more than 4 million people have died (World Health Organization, 2021) [3]. During this period, the WHO advised people to maintain social distance, use masks, to avoid public places such as restaurants, sports clubs, shops and stores, etc., and to cooperate with the WHO and local governments to avoid escalation of this disease [3]. As the coronavirus intensified, people were instructed to leave their home for very limited purposes, such as shopping for food or seeking medical attention. As a result, most sports venues and clubs were closed [4]. In other words, the reduction of physical interaction, known as social or physical distance, has been considered as the first line of defence against this pandemic [5]. However, such social distances, or even temporary quarantines, can themselves have adverse effects on other facets of individuals' lives: such as their physical activity and mental health [6].

Iran is also one of the countries deeply affected by the COVID-19 pandemic. According to the WHO, by the end of July 2021, about 3,623,840 (4.37% of the whole Iranian population) cases have been affected by coronavirus and 88,066 (0.10%) persons have died in Iran [3]. Despite this situation, by the end of July 2021, just over 2,268,826 (2.73%) people had been vaccinated [7]. By this severe spread of the virus in Iran and low pace of vaccination in this country, as well as social and physical restrictions and temporary quarantines, it seems that this country has been severely faced with the subsequent physical and psychological consequences of this pandemic [3]. Undoubtedly, the COVID-19 pandemic has had several effects on individuals’ behaviours [8].

According to reports and researches, critical situations and pandemics may affect differently the physical activity and mental health of people at different levels of society such as patients, health care workers, families, children, students, and even athletes [2, 5, 9, 10]. For example, when a negative event occurs in life, states of anxiety and stress perception are exacerbated by a lack of relationship with others due to social distance [11]. In other words, the current emergency situation that combines both the stress due to COVID-19 with social isolation may have an important emotional and physical impact [12, 13].

The positive effects of regular Physical activity on many health outcomes have been well established [14], and studies have observed this link during the COVID-19 [9]. Indeed, Physical activity has played an important role in reducing fatal outcomes of COVID-19 and served as a good strategy for recovering these patients after being disgorged from the hospital [15]. In addition, exercising regularly and maintaining a lifestyle during quarantine is crucial to prevent future chronic illnesses due to sedentary habits [5]. However, research has shown that engagement in physical activity may have decreased during the COVID-19 pandemic [6, 16]. For example, Amini et al. [16] reported that the physical activity level of Iranian people decreased during the COVID-19 pandemic.

Furthermore, concerning emotional factors, researchers have also shown that quarantine and isolation can also lead to anxiety [17–19]. One of these types of anxiety variables is social physical anxiety. Social physical anxiety is a social psychological variable that is influenced by theories of self-expression and management and it assesses a person's perceived anxiety about presenting his body and figure when they are in front of others [20]. Social physical anxiety is mainly associated with some of the prominent psychological and behavioural factors associated with health. For example, social physical anxiety is associated with variables such as physical self-esteem [21], body image [22] and levels of physical activity [23]. Social physical anxiety like other types of anxieties appears to increase during the COVID-19 pandemic [23, 24]. In addition to this variable, self-esteem as another psychological variable can be affected in this situation [26]. Self-esteem can be defined as person's self- assessment [27]. This variable is important for a successful and satisfying life and is an essential aspect of psychological well-being. Research has shown a positive relationship between physical activity and individual’s self-esteem [28]. In the present study, we tried to pay attention to these physical and psychological variables and evaluate the impact of the COVID-19 pandemic on them. To date, however, much research has been conducted worldwide on the impact of the COVID-19 pandemic on levels of physical activity and mental health [6, 29–31]. Also, to our knowledge, there is little information about this, especially for Iran [32–34]. Therefore, this study is designed to achieve the following two goals. First, this study aimed to compare the level of physical activity and other psychological indicators such as self-esteem and social physical anxiety before (i.e., retrospectively assessed) and during the COVID-19 pandemic. This procedure will give us a better understanding of the adverse effects of pandemics on physical activity and psychological states in Iran. In the second step, this study aimed to examine how changes in physical activity are associated with mental health indicators such as self-esteem and social physical anxiety before and during the COVID-19 pandemic.

## Materials and methods

### Study design and setting

The present study was a cross-sectional web-based study and was performed on a cohort group of Iranian citizen in the spring of 2021. In this study, participants answered the web-based questionnaires of physical activity, self-esteem and social physique anxiety. In these electronic questionnaires, individuals were asked to answer questions in two time periods before and during the pandemic.

### Participants

The sample size was calculated using G-Power 3.1 and according to the significance level of 0.05, statistical power of 0.95 and medium effect size of 0. 3 for examination of correlation between dependent variables, results reported a sample size of 134 participants. However, in this research, an attempt was made to use a higher sample size to increase the power of the test as well as to decrease the possible loss of respondents. Participants were 335 Iranian citizen with a mean age of 30.06 ± 14.58 years. They were selected through convenience sampling from different parts of Iran mainly through virtual social networks (e.g., Instagram, Telegram and WhatsApp) and filled out an online questionnaire after expressing their consent to participate and they had the option to withdraw from the study at any time. They were from different parts of Iran. The eligibility criteria for this study were as follows; 1- Having at least 10 years of age. 2- Having a minimum literacy rate (It means people who could easily read and complete online questionnaires) 3- Ability to work with networks of smart communication devices such as smartphones and computers 4- Living in Iran for the past 5 years.

### Scales

#### The international physical activity questionnaire (IPAQ; short form)

The IPAQ was used to measure participants' physical activity [35]. This questionnaire is designed to be used among adults aged 18 to 65 years [36]. The way of scoring is that activities such as aerobics, high-speed cycling, mountaineering and basketball, which require more than 6 cal per minutes of energy, are classified as intense activities. Activities such as volleyball, badminton, and room cleaning, which require 3 to 6 cal per minute, are classified as moderate activities. Any activity that is less than ten minute is not considered [35]. Scores for sitting time during the week are calculated separately and are not included in the final score estimate. Each Metabolic Equivalent Task (MET) minutes per week represents the amount of energy consumed for physical activities per week. To obtain the continuous variable score of the IPAQ, it is sufficient to multiply the number of days in the time related to physical activity, as well as the number of METs per week to calculate the amount of energy consumed for physical activity. Since this tool measures physical activity in three categories: low activity level, moderate activity level and intense activity level, each of the mentioned activity intensities will have a different weight. For example, the weight of walking will be 3.3, the weight of moderate activity will be 4 and the weight of intense activity will be 6. The final score of physical activity is the sum of scores related to the three categories of low activity level, moderate activity level, and intense activity level [37]. We have used Persian version of this questionnaire which has been used in various studies in Iran and its validity and reliability have been confirmed [16, 38].

### Self-esteem scale

The Rosenberg self-esteem scale (1965) measures a person's overall self-esteem [39]. This scale includes 10 general terms that measures the level of life satisfaction and a good feeling about oneself; for example, the first question was as follows: "On the whole, I am satisfied with myself". Participants had to answer this question using the 4-point Likert scale, from strongly agree to strongly disagree. This tool is a common and valid tool for measuring self-esteem in all over of the world. This scale has a higher correlation than the Coopersmith self-esteem questionnaire and has a higher validity in measuring self-esteem levels [40]. In another study, the internal consistency of this scale was reported to be appropriate and about 0.74 [28]. Cronbach's alpha was observed 0.86 in the current sample. We have used Persian version of this questionnaire which has been used in various studies in Iran and its validity and reliability have been confirmed [41].

### Social physical anxiety scale

The social physical anxiety scale [42] consists of seven items, for example: "I sometimes get upset because I think others judge my weight or fitness negatively. In response to each question, participants use the 5-point Likert scale as: 1 (never) to 5 (always). Higher scores indicate higher levels of social physical anxiety. However, question number 5: I feel empowered about how others evaluate my body; it is scored in reverse [43]. Sáenz-Alvarez et al. [43] reported the validity and reliability of this tool as appropriate. The researchers reported that the internal consistency of the instrument was about 0.85 via Cronbach's alpha. Cronbach's alpha was observed 0.81 in the current sample. We have used Persian version of this questionnaire which has been used in various studies in Iran and its validity and reliability have been confirmed [44].

### Procedure

After approving the research plan in the ethics committee in the research of the department of motor behaviour and sports psychology of Shahid Chamran University of Ahvaz (16,032,021), the researchers designed an electronic questionnaire in the form of Google Form online survey. The time to complete the questionnaire was about 10 min. The online questionnaire consisted of four parts. In the first part, demographic information such as age, gender, level of education, marital status and employment status were asked. In the second part, there were questions related to the IPAQ. In the third part, questions about the participants' self-esteem were asked, and at the end, there were questions related to the social physique anxiety scale. The questionnaire was provided to the participants in the spring (May 1st) of 2021 for 15 days and they were asked to answer the mentioned questions in two periods before the outbreak of coronavirus (winter 2020, March 20st) and the period during the outbreak of coronavirus (spring 2021, May 1st). Finally, 335 questionnaires were accepted among the answered and complete questionnaires and entered the final analysis.

### Data analysis

Statistical analysis was performed using SPSS software version 26. To analyse the data collected through questionnaires, first, preliminary analyses were conducted, such as testing for normality using the Kolmogorov–Smirnov test, and then descriptive statistics were used to describe the data extracted from the questionnaires. According to the purpose of the study, initially the collected data were compared in the two time periods before and during the outbreak of coronavirus. This comparison was performed in both gender of men and women in general. Considering that another purpose of the present study was to investigate the relationship between changes in physical activity and mental health indicators from before the pandemic to the during of it, the residualized change scores were obtained by regressing scored variables during pandemic (e.g., physical activity during the pandemic) on their respective variables before pandemic (e.g., physical activity before the pandemic) [45].

## Results

### Characteristics of participants

The results of descriptive statistics showed that, 155 participants were men (46.26% with a mean age of 33.70 ± 14.72 years) and 180 participants were women (53.74% with a mean age of 26.92 ± 13.74 years), most of participants were single (200 participants, 59.07%) and the rest were married (135 people, 40.03%). Almost most of them (211 participants, 63%) had a university degree. The rest of the participants (124 participants, 37%) had a high school diploma. One hundred twelve (33.4%) of the respondents were students, 132 (39.4%) were employees, and the rest of the participants were employed in freelance and private jobs (91 participants, 27.1%).

### Main results

#### Physical activity and mental health difference before and during the pandemic

**Fig. 1:**
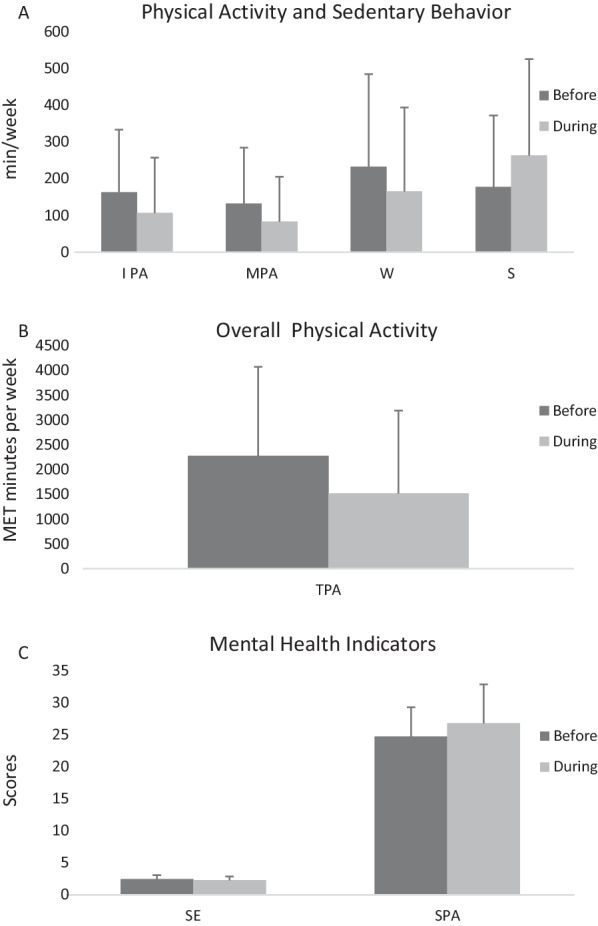
**a** Physical activity indicators such as intense physical activity (IPA; min/week), medium physical activity (MPA; min/week), waking (W; min/week), and also sedentary indicator means sitting (S; min/week), **b** overall physical activity (OPA; MET minutes per week), **c** mental health indicators such as self-esteem (SE) and social physique anxiety (SPA). Error bars represent standard deviation

Preliminary findings also revealed that the extracted data from the questionnaires did not follow a normal distribution; therefore, the researchers used non-parametric statistics to analyse the data. Wilcoxon test was first used to compare the groups in the two time periods before and during the pandemic. The results of this test showed that the participants of the present study reported a significantly lower overall level of physical activity and self-esteem from before to during the COVID-19. These differences were consistent in both genders (Table 1 and Fig. 1).

**Table 1:** Wilcoxon test results for comparison of research variables in two time periods before and during coronavirus pandemic

Variables								
Gender	Statistics	Intense PA (min/week)	Moderate PA(min/week)	Walking (min/week)	Sitting (min/week)	Overal PA	Self-esteem	Physical-social anxiety
Female	Z	− 4.29	− 5.05	− 4.25	− 7	− 5.51	− 6.47	− 6.14
	Significance	0.0001 *	0.0001 *	0.0001 *	0.0001 *	0.0001 *	0.0001 *	0.0001 *
	Effect size (r)	0.23	0.27	0.23	0.38	0.30	0.35	0.33
Male	Z	− 6.05	− 5.67	− 4.87	− 7.62	− 6.67	− 3.47	− 8.11
	Significance	0.0001 *	0.0001 *	0.0001 *	0.0001 *	0.0001 *	0.0001 *	0.0001 *
	Effect size (r)	0.33	0.30	0.26	0.41	0.36	0.18	0.44
Total	Z	− 7.33	− 7.57	− 6.41	− 10.35	− 6.41	− 7.28	− 9.98
	Significance	0.0001 *	0.0001 *	0.0001 *	0.0001 *	0.0001 *	0.0001 *	0.0001 *
	Effect size (r)	0.40	0.41	0.35	0.56	0.35	0.39	0.54

*PA* physical activity

^*^P < 0.05

#### Relationship between physical activity and mental health

The results of Spearman correlation test indicated a significant negative relationship between self-esteem and social physical anxiety changes from before to during of COVID-19 pandemic. This significant negative relationship was observed in both genders. However, the results did not show any significant association between changes in physical activity and in social physical anxiety as well as with changes in self-esteem, from before and during coronavirus (Table 2 and Fig. 1).

**Table 2:** Results of Spearman rank correlation test to investigate the relationship between changes in variables, based on residualized scores

	Gender	Research variables	Over-all PA (day*min*MET)	Self-esteem
Changes scores (residualized scores) before and during the pandemic	Females	Over-all PA (day*min*MET)	–	–
		Self-esteem	− 0.055[95% CI − 0.16;0.05]	–
		Physical-social anxiety	0.107[95% CI − 0.01; 0.21]	− 0.369**[95% CI − 0.46; − 0.27]
	Males	Over-all PA (day*min*MET)	–	–
		Self-esteem	− 0.149[95% CI − 0.25; − 0.04]	–
		Physical-social anxiety	0.019[95% CI − 0.09; 0.13]	− 0.285**[95% CI − 0.38; − 0.18]
	Total	Over-all PA (day*min*MET)	–	–
		Self-esteem	− 0.095[95% CI − 0.02; 0.01]	–
		Physical-social anxiety	0.083[95% CI − 0.02; 0.19]	− 0.337**[95% CI − 0.43; − 0.24]

*PA* physical activity

**P < 0.01

## Discussion

To our knowledge, this study is one of the first studies in Iran that not only examines the impact of the COVID-19 pandemic on the level of physical activity, but also examines other mental health indicators such as self-esteem and social physical anxiety during the COVID-19 pandemic. Specifically, this study had two objectives. First, it aims of to compare physical activity levels, self-esteem and social physical anxiety of the participants from before to during the COVID-19 pandemic. Second, it aims to investigate the relationship between changes in these variables, from before to during the COVID-19 pandemic.

For first aim of the present study, as predicted, the results showed that all the measured indices were different before and during the pandemic, lower levels of physical activity and self-esteem and higher levels of social anxiety from before to during the COVID-19. The findings indicating detrimental effects of the COVID-19 pandemic on the levels of physical activity and of psychological indicators are in line with previous studies [6, 15, 29–31, 33, 34]. For example, Di Franco et al. [10] in their study examined the effects of social distance on mental health status and physical activity during the COVID-19 pandemic. In their study of 1,132 Italian athletes, the researchers concluded that the COVID-19 pandemic had a negative effect on perceived stress as well as psycho-biological and social conditions.

Amini et al. [16], in another research, show that the COVID-19 pandemic negatively affected the level of physical activity of Iranian individuals. Therefore, the findings of the present study, in line with the findings of Amini et al. [16], show that the level of physical activity of the Iranian people has decreased significantly compared to the time before the pandemic. The results of this study also showed that the level of sedentary behaviours (the amount of time people spend sitting) has increased significantly. These changes are seen in both genders. Amini et al. [16] also observed the negative effects of pandemics in both genders. However, others have shown that the pandemic has mainly affected the intensity of physical activity and not its frequency in Iran [32]. Since, research has also shown that physical activity is associated with decreased hospitalisation and can significantly reduce the mortality rates of patients with COVID-19 [Rahmati], and also considering that it has been determined, physical activity can bring many benefits, including improving the levels of well-being and sleep quality as well as reducing people anxiety [33, 34]. Therefore, it is suggested that people, especially the older adults try to increase their resistance to various diseases, including COVID-19 pandemic, by increasing their level of physical activity throughout their lives [46, 47].

For second aim of the present study and contrary to our hypothesis, we only found one significant negative association between self-esteem of individuals and their physical social anxiety (higher self-esteem was associated with lower physical social anxiety) both on the changes scores from before to during the COVID-19. However, physical activity changes were associated neither with self-esteem nor with physical social anxiety changes.

There are several related studies in this field, for example, Cheval et al. [9] examined the association between physical activity change, sedentary behaviour, and mental health during the coronavirus pandemic in France and Switzerland. In their study, participants also answered online questionnaires that measured physical activity, mental and physical health, anxiety and depressive symptoms. Their findings show that the COVID-19 pandemic, although leading to a decrease in vigorous activity as well as an increase in sedentary behaviour among the population, the amount of moderate physical activity or walking among individuals have also increased. In the present study, physical activity in all its indicators such as moderate and vigorous physical activity, walking and over-all physical activity score decreased during the COVID-19 pandemic period compared to before. The findings of the present study are consistent with the findings reported by Cheval et al. [9] regarding vigorous physical activity, but the results are in contrast with their findings on other indicators such as moderate physical activity and walking. These differences can be explained considering the culture of sports and physical activity among the participants. We can speculate that people in developed countries such as France and Switzerland are more likely than people in countries like Iran to employ benefits of physical activity to improve their own physical and psychological health. Another possible reason for the decrease in physical activity of the participants of the present study after the pandemic is the type of occupation of individuals. As reported in the results and participants sections, the majority of those who participated in the present study were either students and university students or government employees. Universities and schools were closed during the COVID-19 pandemic. Virtual and distance education were implemented to replace traditional teaching strategies. In addition, for employees, telecommuting has replaced attendance at work. Overall, it seems that these frequent and long stays at home, as well as the available stress and fear of leaving home, all led to increased sedentary behaviours and less presence in sports places.

Although the results of the present study did not show any significant relationship between physical activity changes and other psychological indicators such as self-esteem and social physical anxiety changes, they indicated a significant and negative relationship between self-esteem and social physical anxiety. To explain this finding, it can be said that high self-esteem has probably reduced concerns about weight and appearance of people in social situations and therefore has reduced the level of social physical anxiety [21]. This relationship has been established in this study.

In the present study, decreased self-esteem and increased post-pandemic social physique anxiety were reported. Research has shown that with the onset of the COVID-19 pandemic, people's use of social networks such as Instagram has increased dramatically [48]. On the other hand, it has been found that there is a relationship between the use of social networks and increased body dissatisfaction and reduced self-esteem [48]. Therefore, the reported decrease in self-esteem, as well as the increase in levels of social physique anxiety among the sample studied in the present study, can be attributed to the possible increase in people's use of social networks, especially Instagram. In the present study, the use of social networks has not been measured. It is suggested that future research measures the use of social networks during the pandemic and compares it with before the pandemic, to examine the impact of the pandemic address the level of physical activity and indicators of mental health such as self-esteem and social physique anxiety.

Physical activity is an essential part of a healthy lifestyle that can prevent or manage chronic diseases, facilitate daily life activities, and also maintain physical function in people, especially the elderly [9]. In other words, physical activity can be associated with a myriad of positive physical and mental health consequences [9]. However, since the results of the present study could not show a relationship between the level of physical activity and other psychological variables, it is suggested that the findings of this study should be applied more cautiously. Therefore, governments especially the government of Iran, with the help of experts in the field of sports and health should create conditions allowing individuals to participate in sports and physical activity with more motivation and facilities. They could also aim to prevent the accumulation of sedentary behaviours, which are associated with detrimental mental and physical problems.

To our knowledge, this study is one of the first studies in an Iranian sample which investigates the impact of the COVID-19 pandemic on the level of physical activity and on some psychological variables. However, the results of the present study have several potential limitations. First, the study results are limited by the traditional limitations of cross-sectional study designs. For example, one of the limitations of the present study was the retrospective assessment of variables in reference of the before-lockdown period. In this study, participants were asked to report their level of physical activity and psychological indicators in the two time periods before and during the COVID-19 pandemic. Although post-pandemic questions coincided with research implementation, pre-pandemic questions were retrospective and past-related. The next limitation of the present study can be attributed to the geographical scope of the respondents who completed the web-based questionnaire. This dispersion may not be the same throughout the country in different regions and may be more specific to a particular part of the country (In the present study, participants were from different cities of Iran, but most of them were from Khuzestan, Tehran, Alborz and Kurdistan provinces). Another limitation of the present study was that it was a web-based one. Mainly, some people could not participate in this study due to lack of Internet access, absence in cyberspace or lack of knowledge to complete the questionnaires of this study. In addition, due to the fact that the international physical activity questionnaire is designed for people over 18 years old, and in the present research, some people completed this questionnaire at a younger age, so this is another limitation of the current research and it should be to be considered in future research. Also, since the sampling method used in this research is a convenience sampling, which is one of the non-random sampling methods and have lower external validity than other random methods, it is suggested to use the results of this research with more caution. Due to the above-mentioned limitations, it is suggested to conduct other researches, which may tackle those limitations.

## Conclusions

The findings of this study showed that the pandemic was associated with lower the levels of physical activity among the Iranian people, and with lower self-esteem and higher physical social anxiety. However, unlike previous studies, we did not observe a significant association of physical activity with self-esteem and social physical anxiety. Future large-scale studies and with a more heterogeneous sample of Iranian people should be conducted to further examine the expected protective role of physical activity on mental health outcomes. Meanwhile, public policies aiming to buffer the negative impact on COVID-19 Iranians’ health are urgently needed.

## Data Availability

The datasets generated and analyzed during the current study are not publicly available due to ethical restrictions, however, they are available from the corresponding author on reasonable request.
